# Biomechanical effect of combined use of mini-screws and aligners in preventing uncontrolled tipping of the incisor and mesial tipping of the molar: a three-dimensional finite element study

**DOI:** 10.1186/s40510-025-00601-2

**Published:** 2025-12-29

**Authors:** Runa Yamaguchi-Higuchi, Ryo Hamanaka, Hiroya Komaki, Toshiro Emori, Jun-ya Tominaga, Yui Horiguchi, Sayaka Iwata, Kazuhiro Ogawa, Arina Kitaura, Noriaki Yoshida

**Affiliations:** 1 Nagasaki University Graduate School of Biomedical Sciences, Nagasaki, Japan; 2 Nagasaki University Hospital, Nagasaki, Japan

**Keywords:** Clear aligners, Finite element method, Mini-screws, Elastics

## Abstract

**Background:**

This study aimed to evaluate the effect of external force applied from mini-screws to the aligner during space closure in extraction cases, and to determine the optimal loading conditions to prevent lingual crown tipping of the incisor and mesial tipping of the molar using the finite element (FE) method.

**Methodology:**

A three-dimensional FE model of the maxillary dentition with extraction of first premolars was constructed, and three different loading conditions were designed. The aligner was activated for 0.25 mm of incisor retraction without using mini-screws, with application of 0, 100, 200, or 300 cN of distal force through mini-screws placed in the posterior region, or with application of 0, 100, 200, 300, or 400 cN of intrusive force through mini-screws placed in the anterior region, in addition to the application of 300 cN of distal force.

**Results:**

As the magnitude of distal force increased, the degree of mesial tipping of the first molar gradually decreased, becoming almost zero with nearly 300 cN of distal force. As the magnitude of intrusive force increased in addition to the application of 300 cN of distal force, the degree of lingual crown tipping of the central incisor gradually decreased, becoming almost zero with nearly 400 cN of intrusive force.

**Conclusion:**

Application of 300 cN of distal force to the aligner from mini-screws placed in the posterior region could completely prevent mesial tipping of the molar and anchorage loss during anterior retraction. Application of 400 cN of intrusive force from mini-screws placed in the anterior region could prevent lingual crown tipping of the incisor, thus providing better torque control for the incisors.

## Introduction

Aligner treatment is becoming increasingly popular among adult patients seeking orthodontic treatment because of the esthetic and comfort advantages over fixed orthodontic appliances [1]. Despite the fact that aligners are used worldwide, the mechanisms of force transmission involved are still not fully understood. The tooth movements achieved with aligners thus differ from those planned in the virtual setup [2]. Aligner treatment is quite efficient in leveling anterior teeth and achieving molar distalization, but is less efficient in correcting deep overbite, controlling root movement, or expanding the dentition [3–6]. Mesial tipping of molars and an increased anterior overbite sometimes occur in patients with extracted premolars [7–9]. Aligner treatment has therefore been considered more advantageous for non-extraction cases, but not for complex extraction cases requiring a high level of skill [10]. Previous studies have reported that tipping movement is more likely to occur, whereas achieving bodily movement in extraction cases is extremely difficult with aligners [1, 2]. Clinical reports have indicated that molars tend to show excessive mesial tipping along with anchorage loss, and incisors show uncontrolled tipping [11] as well as extrusion, which may cause premature contact between the upper and lower anterior teeth as undesirable side effects [12].

The use of mini-screws in conjunction with aligner treatment could overcome these drawbacks [13]. Lin et al. [14] demonstrated treatment mechanics that simultaneously prevent uncontrolled tipping and extrusion of the incisor and mesial tipping of the molar using mini-screws placed in the anterior and posterior regions. They suggested that the application of distal force to an aligner from mini-screws placed in the posterior region could suppress anchorage loss, thereby preventing molars from tipping mesially during space closure in extraction cases. In addition, the application of intrusive force from mini-screws placed in the anterior region could provide better torque control of incisors and prevent their extrusion. Nevertheless, the amount of force required on the mini-screws to prevent mesial molar tipping and to achieve controlled tipping or bodily movement of the incisors using the above-mentioned mechanics is still unknown.

Several studies [15–17] have been conducted to investigate the mechanical behavior of aligners and the resultant tooth movements using the finite element (FE) method. However, very few attempts have been made to propose methods for improving the therapeutic efficiency of aligners. The purpose of this study was to determine the optimal loading conditions and appliance design that can prevent inherent side effects of aligner treatment and provide highly predictable tooth movement using the FE method.

## Materials and methods

**Fig. 1:**
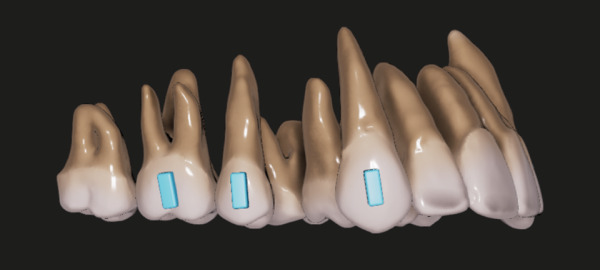
Three-dimensional model of maxillary dentition in which the first premolars are extracted. Vertical rectangular attachments (4.0 mm high, 1.5 mm wide, and 1.0 mm thick) were placed on the canine, second premolar, and first molar

A three-dimensional (3D) scan of a typodont model (E50-500 A; Nissin Dental Products, Kyoto, Japan) with normal occlusion was used to create a 3D FE model of the maxillary dentition with the first premolar extracted. Teeth were modeled using rigid elements [18–20]. The periodontal ligament was modeled using spring elements with six degrees of freedom according to Kojima et al. [18, 21, 22] and was assumed to be 0.2 mm thick with a Young’s modulus of 0.03 MPa [23–25] and a Poisson’s ratio of 0.3. Aligners were modeled using shell elements, and vertical rectangular attachments (4.0 mm high, 1.5 mm wide, and 1.0 mm thick) were placed on the buccal surfaces of the canine, second premolar, and first molar, respectively (Fig. 1). Young’s modulus and Poisson’s ratio of the aligner were set at 700 MPa and 0.3, respectively, and contact boundary condition was prescribed between tooth surface and inner surface of the aligner using the gap elements.

The loading condition, wherein the aligner was activated for 0.25 mm of space closure, was simulated in the following steps. First, the anterior teeth were displaced in the model of maxillary dentition in the palatal direction by 0.25 mm on the assumption that they were retracted bodily. Then, an aligner model with a uniform thickness of 0.75 mm was constructed on the surface of each tooth in the dentition model after a reduction in extraction space of 0.25 mm. In the next step, each node forming the surface of the anterior teeth of the model was displaced in the labial direction by 0.25 mm. Although the procedure of loading in FE analysis is the opposite of the clinical procedure, both are mechanically equivalent and this method is widely used [26–30].

**Fig. 2:**
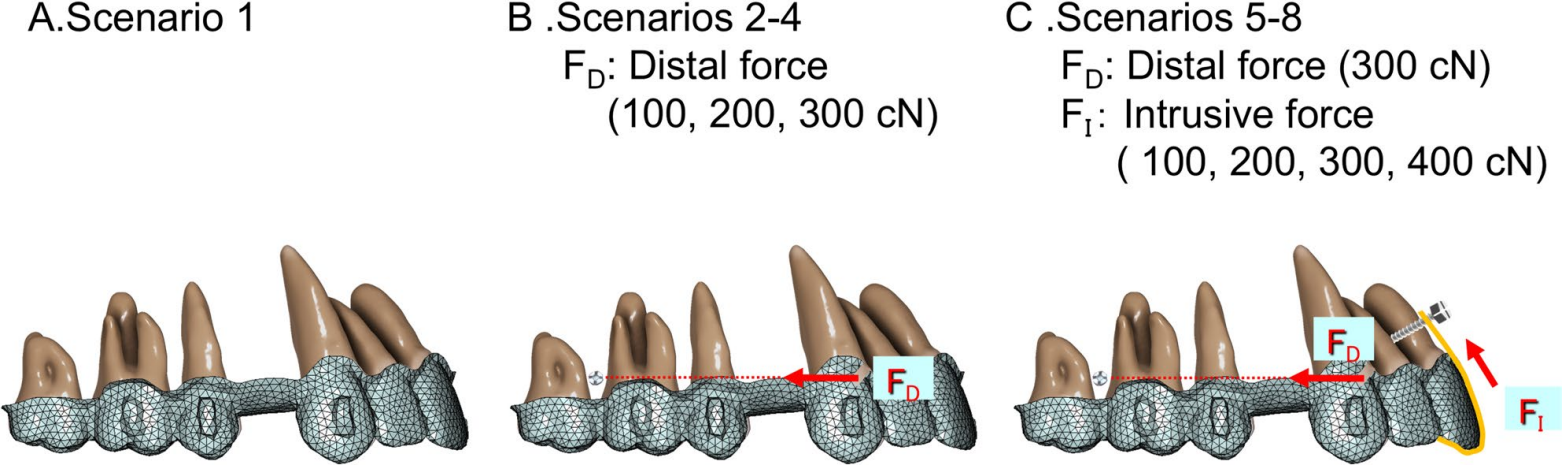
Three-dimensional FE model of the aligner placed on the maxillary dentition. Eight loading conditions are shown Scenario 1: The aligner is activated for incisor retraction of 0.25 mm without mini**-**screws. Scenario 2: Posterior mini**-**screws with a distal force of 100 cN applied to the canine hook. Scenario 3: Posterior mini**-**screws with a distal force of 200 cN applied to the canine hook. Scenario 4: Posterior mini**-**screws with a distal force of 300 cN applied to the canine hook. Scenario 5: Anterior mini**-**screws with an intrusive force of 100 cN applied to the incisors, combined with a distal force of 300 cN from posterior mini**-**screws. Scenario 6: Anterior mini**-**screws with an intrusive force of 200 cN applied to the incisors, combined with a distal force of 300 cN from posterior mini**-**screws. Scenario 7: Anterior mini**-**screws with an intrusive force of 300 cN applied to the incisors, combined with a distal force of 300 cN from posterior mini**-**screws. Scenario 8: Anterior mini**-**screws with an intrusive force of 400 cN applied to the incisors, combined with a distal force of 300 cN from posterior mini**-**screws

The forces were directed toward mini-screws on the assumption that they were placed between the second premolar and the first molar at a position 7 mm apical to the alveolar crest on the buccal when applying distal forces, and between the central and lateral incisors at a position 5 mm apical to the alveolar crest on the labial side when applying intrusive forces. The overall configuration of mini-screw placement and force application is illustrated in Fig. 2.

In the first scenario, the aligner was activated for incisor retraction of 0.25 mm without applying external forces from mini-screws (Fig. 2A). The second, third, and fourth scenarios assumed distal forces of 100, 200, and 300 cN, respectively, produced by elastics from posterior mini-screws to the canine hook, representing a direct anchorage approach to prevent mesial tipping of the first molar during aligner activation (Fig. 2B). The fifth, sixth, seventh, and eighth scenarios assumed intrusive forces of 100, 200, 300, and 400 cN, respectively, applied to the incisors from anterior mini-screws through lingual aligner cuts, in combination with a distal force of 300 cN from posterior mini-screws to the canine hook (Fig. 2C). In this setting, intrusive force was applied to the incisors by applying elastics to the mini-screws placed between the central and lateral incisors to prevent lingual crown tipping of the incisors. The magnitude of intrusive force was set at 100, 200, 300, or 400 cN.

Displacement of each tooth under the application of intrusive and distal force from the mini-screws was analyzed using the FE method. FE analysis was performed using an FEM package (Marc 2017.1; MSC Software, Los Angeles, CA). The position of the center of resistance of each tooth was determined according to the method previously reported by Hamanaka et al. [19].

## Results

**Fig. 3:**
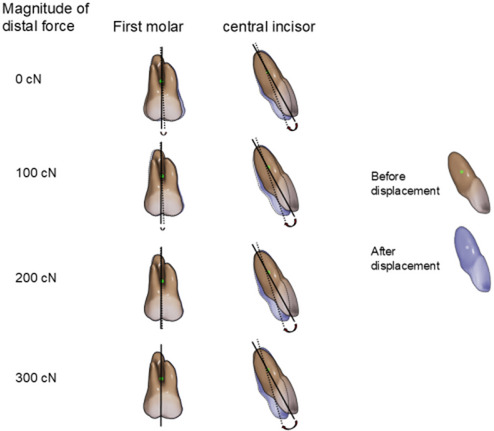
Movement pattern of central incisors and first molars from the sagittal view in scenarios 1–4. Positions of teeth before and after aligner activation are indicated as solid and dotted outlines, respectively. The amount of tooth displacement was magnified 10 times for better understanding

Figure 3 shows the movement patterns of the central incisor and first molar from the sagittal view in Scenarios 2, 3, and 4, including Scenario 1 (without using mini-screws). When the anterior teeth were displaced in the palatal direction by 0.25 mm through aligner activation without applying any external force from mini-screws in Scenario 1, the central incisor showed lingual crown tipping and the first molar tipped significantly mesially. On the other hand, when a distal force of 100, 200, or 300 cN was applied to the canine hook of the aligner from mini-screws placed in the posterior region in addition to the aligner activation in Scenarios 2–4, the tendency of the molar to tip mesially decreased, whereas the tendency of the incisor to tip lingually and to extrude increased with the increment of the force magnitude.

**Fig. 4:**
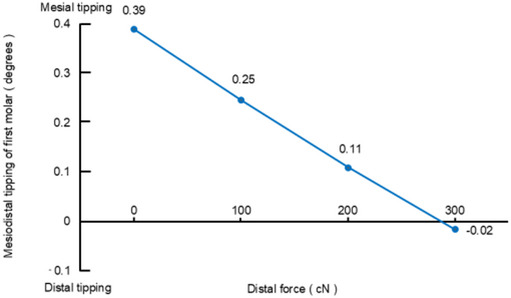
Degree of mesiodistal tipping of the first molar in scenario 2–4. Positive signs indicate mesial tipping, whereas negative signs indicate distal tipping

**Fig. 5:**
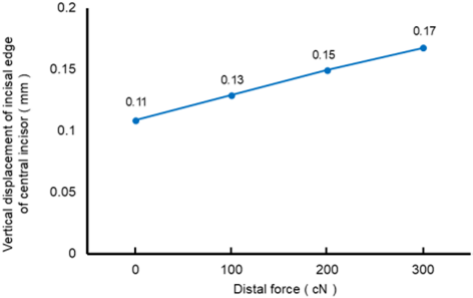
Vertical displacement of the incisal edge of the central incisors in Scenario 2–4 in the direction of extrusion

Figure 4 shows the degree of mesiodistal tipping of the first molar as a function of the magnitude of the distal force in Scenarios 2–4. The degree of mesial tipping of the first molar decreased with increased magnitude of the distal force applied from mini-screws, and became zero when applying force of around 300 cN. Figure 5 shows displacement of the incisal edge of the central incisor in the extrusive direction; that is, the amount of relative extrusion as a function of the magnitude of distal force from mini-screws in Scenarios 2–4. As the magnitude of distal force increased, the amount of extrusion displacement at the incisal edge of the central incisor also increased.

**Fig. 6:**
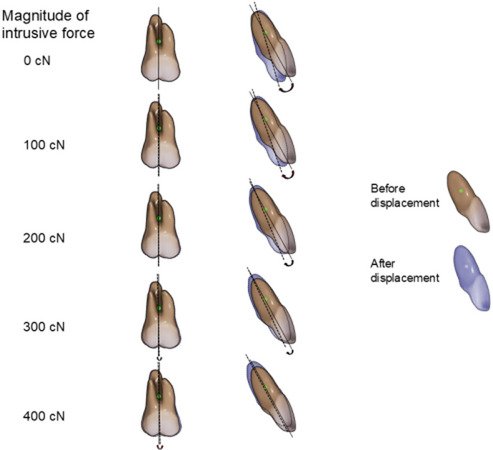
Movement pattern of the central incisor and first molar from the sagittal view in scenario 3–8. Positions of teeth before and after aligner activation are indicated as solid and dotted outlines, respectively

Figure 6 shows the movement pattern of the central incisor and first molar from the sagittal view during incisor retraction of 0.25 mm with application of 300 cN of distal force from mini-screws in the posterior region and additional intrusive force of 100, 200, 300 or 400 cN from mini-screws in the anterior region, respectively (Scenario 3–8). When intrusive force of 100 cN was applied from mini-screws in the anterior region in addition to distal force of 300 cN, the degree of lingual crown tipping and displacement of extrusion at the incisal edge of the central incisor were slightly decreased. When intrusive force was increased from 100 to 200 cN, the degree of lingual crown tipping of the incisor and amount of relative extrusion were further decreased. When intrusive force of 300 cN was applied, the central incisor was obviously intruded and showed a reduced degree of lingual crown tipping. When intrusive force of 400 cN was applied, the incisor was substantially intruded without lingual crown tipping. On the other hand, the tendency for mesial tipping of the first molar increased as the magnitude of intrusive force increased.

**Fig. 7:**
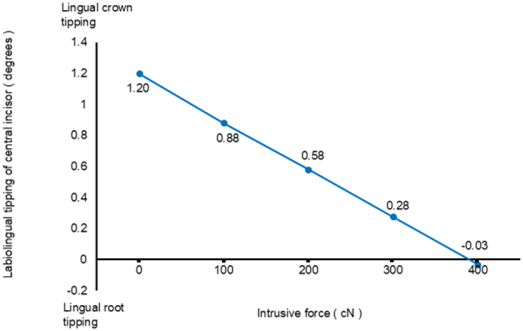
Degree of labiolingual tipping of the central incisor in scenario 3–8

Figure 7 shows the degree of labiolingual tipping of the central incisor as a function of the magnitude of intrusive force in Scenario 3–8. As the magnitude of intrusive force increased, the degree of lingual crown tipping gradually decreased. The amount of relative extrusion of the central incisor became almost zero with the application of 400 cN of intrusive force.

**Fig. 8:**
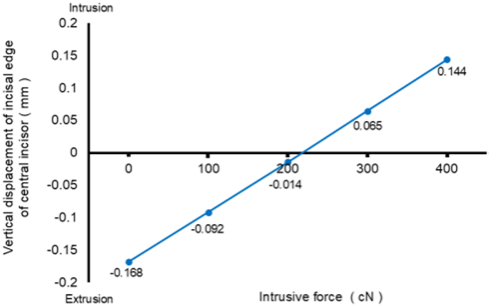
Vertical displacement of central incisors in scenario 3–8

Figure 8 shows the vertical displacement of the incisal edge of the central incisor as a function of the magnitude of intrusive force in Scenario 3–8. As the magnitude of intrusive force increased, the amount of extrusion displacement at the incisal edge of the central incisor decreased. The amount of extrusion became zero when the magnitude of intrusive force exceeded 200 cN. The direction of displacement at the incisal edge then changed from extrusion to intrusion.

## Discussion

Since aligner treatment was introduced to orthodontic practice, this technique has been widely applied to patients desiring esthetic treatment. However, several biomechanical limitations have been identified, particularly in cases involving premolar extraction [11, 12, 31]. Baldwin et al. [32] reported that treatment with aligners in premolar extraction patients was associated with increased tipping of the teeth adjacent to the extraction sites. Simon et al. [33] suggested that the efficacy of incisor torque was almost 50% even with power ridges and that no significant differences in efficacy were seen between the use of conventional attachments and power ridges. An FE study [17] also indicated that incisors showed uncontrolled tipping and extrusion although bodily movement was prescribed during space closure with aligners. According to the study conducted by Dai et al. [12], a greater degree of mesial tipping of the first molar was generated as compared with movements predicted in the simulation. Premolar extraction treatments with aligners are thus challenging because uncontrolled tipping of the incisor and mesial tipping of the molar is quite difficult to prevent. Those side effects may be due to the properties of aligner materials such as a high rate of stress relaxation [34].

Given this situation, it has been suggested that overcorrections or refinements, namely, additional sets of aligners, become necessary to achieve controlled and predictable tooth movements in premolar extraction cases with aligners although such a solution requires a longer treatment period. Another solution to reduce side effects associated with aligner treatment would be to use mini-screws. A clinical report [14] demonstrated that placement of mini-screws in the posterior region could reinforce molar anchorage and prevent the posterior teeth from tipping mesially, and placement of mini-screws in the anterior region could prevent uncontrolled tipping and extrusion of the incisors during space closure. However, no study has determined how much force should be applied to mini-screws to prevent anchorage loss or anterior torque loss and thus provide better controlled movements of both the anterior and posterior segments.

Mini-screws were assumed to be located at a position 7 mm from the alveolar crest for the posterior anchorage, and at a position 5 mm from the alveolar crest for the anterior anchorage. The height level of mini-screws were determined based on data from previous studies as shown below. As for the posterior region, CBCT-based studies have shown that the interradicular spaces between the first and second molars, approximately 6–8 mm from the alveolar crest, provide reliable safe zones [35, 36]. As for the anterior region, it was reported that mini-screws were recommended to be positioned 4–6 mm from the alveolar crest to avoid root contact and optimize force vectors [37, 38].

First, the present study investigated the effect on tooth movement patterns of different magnitudes of distal force applied from mini-screws in the posterior region. In Scenario 1, the first molar demonstrated substantial mesial tipping. On the other hand, the incisor showed marked lingual crown tipping although bodily retraction of the incisors was prescribed in the setup. The mesial tipping of the first molar was reduced when distal force was applied in conjunction with aligner activation in scenario 2–4. As the magnitude of distal force increased, the degree of mesial tipping of the first molar gradually decreased, and became almost zero with the application of nearly 300 cN of distal force. This indicates that distal force of 300 cN could completely prevent anchorage loss as well as mesial tipping of the first molar. In other words, the use of mini-screws could effectively protect the molar anchorage.

Conversely, in Scenario 2–4, the lingual crown tipping and incisor extrusion became more pronounced as the magnitude of the distal force exerted by the mini-screws increased. This is because the incisors receive greater distal force from mini-screws in addition to the retraction force generated by aligner activation. Thus, use of mini-screws placed in the posterior region could avoid mesial tipping of the first molar, whereas lingual crown tipping and extrusion of the incisors are more likely to occur as undesirable side effects. Previous studies have suggested that there is no substantial biomechanical difference between direct and indirect anchorage in terms of posterior anchorage reinforcement [39, 40]. We agree that indirect anchorage management is also a valuable clinical strategy; however, In the present study, we quantified the biomechanics of direct anchorage. Indirect anchorage would have required adjunctive fixed appliances and, consequently, the posterior teeth to be prescribed as non-moving in the setup; therefore, direct anchorage provided a simpler and more transparent framework for our analysis. A previous study [17] suggested that a certain amount of intrusion should be incorporated into the setup during space closure to prevent extrusion of the incisor and the resultant premature contact between the upper and lower anterior teeth.

**Fig. 9:**
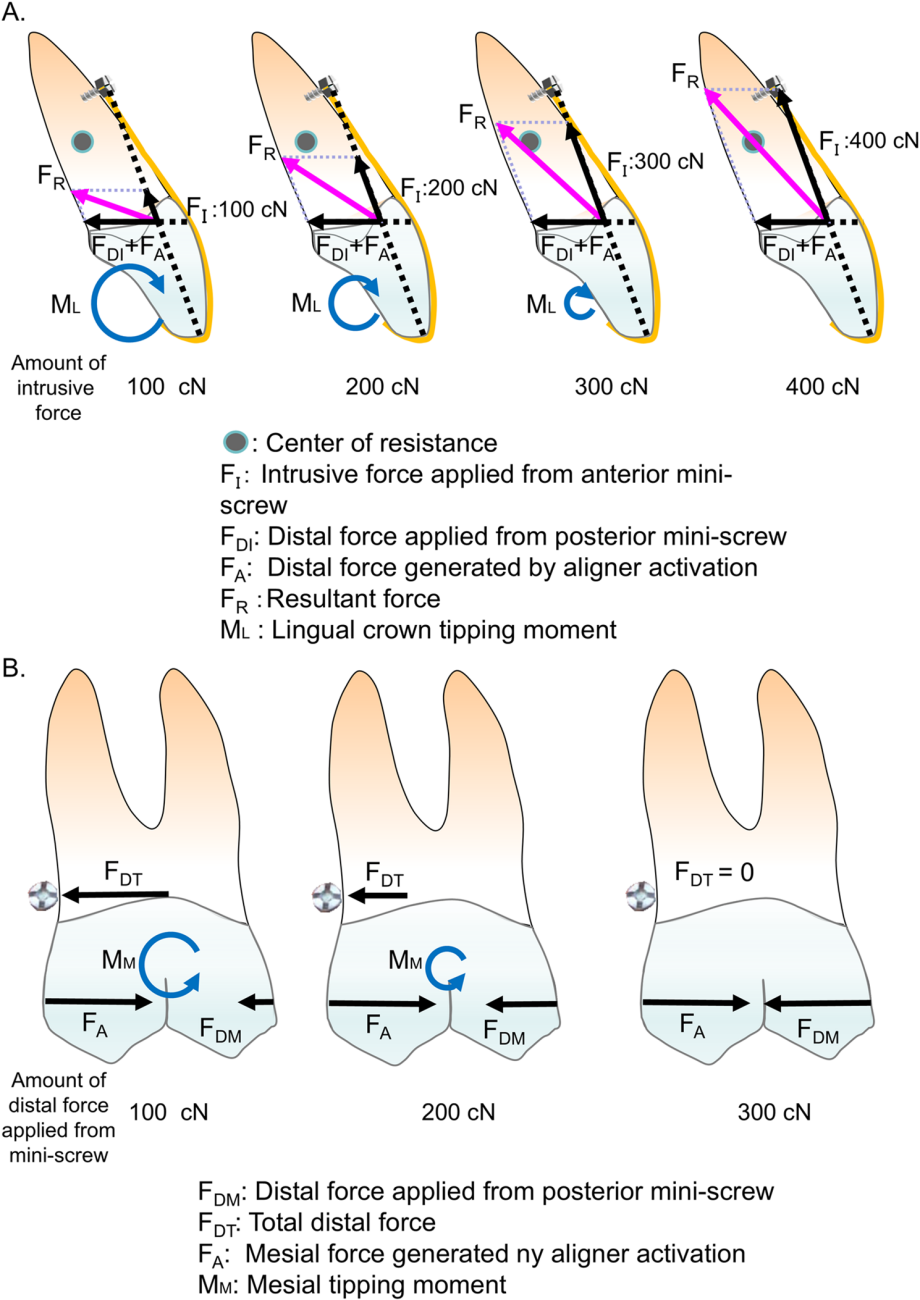
Force systems acting on the maxillary central incisor and first molar when intrusive and distal forces are applied from mini-screws. (A) Anterior region: The intrusive force passes labially to the center of resistance of the central incisor, producing a lingual root tipping (antitipping) moment. (B) Posterior region: The distal force passes coronal to the center of resistance of the first molar, generating a distal tipping moment. Anchorage loss could be prevented when F_DM_ was applied at the same magnitude as F_A_

Lin et al. [14] presented treatment mechanics that can apply intrusive force in addition to distal force to the aligner from mini-screws respectively placed in the anterior and posterior regions to prevent uncontrolled tipping and extrusion of the incisor as well as mesial tipping of the molar during space closure. Figure 9 shows a force system acting on the central incisor when applying intrusive force to the anterior portion of the aligner from mini-screws placed between the central and lateral incisors. Since the intrusive force passes labially to the center of resistance of the central incisor, a lingual root tipping moment is produced, which could decrease or cancel the effect of lingual crown tipping and prevent extrusion of the incisor. In the posterior region, a mesial force is generated on the first molar due to the 0.25-mm activation of the aligner. However, when the distal force from the posterior mini-screws exceeds this mesial force, a resultant force acts coronal to the center of resistance of the first molar, thereby producing a distal tipping moment.

We then investigated the effects on tooth movement pattern of different magnitudes of intrusive force applied in addition to 300 cN of distal force. In scenario 7, simultaneous application of 300 cN intrusive and distal forces and 0.25 mm activation of the aligner successfully reduced the degree of lingual crown tipping of the central incisor. As the magnitude of intrusive force was increased in addition to the application of 300 cN of distal force, the degree of lingual crown tipping of the central incisor gradually decreased, becoming almost zero on application of nearly 400 cN of intrusive force. That is, the application of intrusive force provided better torque control of the incisor, and bodily retraction would be achieved on applying intrusive force of 400 cN in addition to distal force of 300 cN. At this time, the central incisor intruded to a considerable extent.

We also investigated the effect of intrusive force on incisor movement in the vertical dimension by focusing on displacement of the incisal edge. The incisor showed marked extrusion without adding intrusive force, which would increase the risk of premature contact between upper and lower incisors. In Scenarios 3–8, as the intrusive force increased, the displacement associated with the extrusion of the incisal edge decreased and reached zero in Scenario 6 (200 cN). In other words, the incisal edge of the central incisor was not extruded, but maintained its vertical position with the application of 200 cN of intrusive force. When the magnitude of intrusive force exceeded 200 cN, the incisal edge was displaced in the intrusive direction. Thus, application of intrusive force of 200 cN was suggested to completely prevent the bite from deepening; that is, avoiding premature contact in the anterior region. The application of over 200 cN of intrusive force would intrude the position of the incisal edge, resulting in the correction of gummy smile. However, the tendency for mesial tipping of the first molar increased as the magnitude of intrusive force increased. We have to keep in mind that as we try to achieve more effective torque control of the anterior teeth, more anchorage loss will be produced in what is known as the rowboat effect [41].

The present study suggested that application of 300 cN of distal force and 400 cN of intrusive force from mini-screws could minimize the tipping tendencies of both anterior and posterior teeth, completely prevent anchorage loss or mesial tipping for the posterior segment, and cancel out lingual crown tipping of the incisors, providing better torque control during space closure in extraction cases. In this balance of external force applied to the aligner, as the combination of 300 cN of distal force and 400 cN of intrusive force, bodily retraction and intrusion of the incisors could be achieved. The prescription of such loading conditions appears indicated for the correction of maxillary protrusion accompanied by gummy smile and normal inclination of the incisor. If there is no gummy smile and controlled tipping rather than bodily movement of the incisor is required, application of 300 cN of distal force combined with 200 cN of intrusive force would be recommended.

As for the physical properties of aligner, previous studies [16, 42–44] reported a considerable range in the values of Young’s modulus and Poisson’s ratio, with the modulus varying from 528 to 2500 MPa and the Poisson’s ratio ranging from 0.3 to 0.49. In the present study, we selected a Young’s modulus of 700 MPa, which reflects the effective stiffness of aligner sheet(Zendura FLX, Bay Materials LLC, Fremont, CA, USA)following approximately 50% stress relaxation, as indicated by recent experimental findings [45].

There are inherent limitations in finite element analysis, as it cannot fully reproduce the biological environment. The mathematical modeling of living tissues and the availability of experimental data are restricted, and at the current level of technology, constructing a model entirely equivalent to a living body is virtually impossible. Moreover, individual variability among patients adds further complexity. Consequently, the results of the present study do not guarantee that the same responses would occur in vivo. Nevertheless, the finite element method has the distinct advantage of high reproducibility. Unlike physical experiments, where replicating identical conditions each time is nearly impossible, computer-based finite element simulations allow 100% reproducibility in the analytical environment. This enables precise evaluation of how variations in loading conditions influence patterns of tooth movement.

Another limitation is that the material properties of aligners are neither uniform across products nor time-invariant during intraoral use. Variations in elastic modulus among polymers, as well as degradation and stress relaxation over time, can influence the absolute level of force delivery. Therefore, the magnitude of forces predicted in this study should be interpreted with caution. In clinical practice, weaker forces may be required if the aligner material is softer or undergoes greater degradation, whereas stronger forces may be necessary if the material is stiffer. Such adjustments are essential to ensure that the mechanical predictions from FE analysis are applied appropriately to patient care.

In the present study, the forces generated under the given simulation conditions were stronger than the levels previously suggested to be ideal. Earlier studies have indicated that the optimal force magnitude for retraction of the anterior segment is around 250 g, and that the total force should remain light and not exceed 300 g [46, 47]. Our results, however, showed that a 0.25-mm activation of the aligner in the early phase of space closure could produce a retraction force of about 300 cN acting on the anterior teeth. Such force levels may lead to undesirable side effects, including root resorption, loosening of mini-screws, or patient discomfort. Therefore, regular clinical monitoring is essential.

Future studies incorporating patient-specific models and biological variability are thus essential to validate and refine these findings for clinical application. Furthermore, it is necessary to verify the validity of FE analysis by comparing the results obtained from clinical studies.

## Conclusions

The closure of premolar extraction spaces with clear aligners remains a clinical challenge, particularly due to the risks of anchorage loss and uncontrolled tipping. This FE study elucidated the biomechanical effects of mini-screw–assisted aligner mechanics in extraction cases and provides practical guidelines that can be tailored to specific treatment goals:

1. Molar anchorage preservation during anterior retraction.
2. The application of distal force to the canine hook of the aligner from mini-screws placed in the posterior region could reduce mesial tipping of the molar. However, this approach may increase lingual crown tipping and extrusion of the incisors.
3. Torque control of the maxillary incisors without bodily intrusion.
4. The application of 300 cN distal force and 200 cN intrusive force from mini-screws appears indicated to correct maxillary protrusion without gummy smile in cases where controlled tipping rather than bodily movement is required.
5. Bodily retraction with incisor intrusion.
6. The application of 300 cN distal force and 400 cN intrusive force from mini-screws appears indicated for the correction of maxillary protrusion accompanied by gummy smile and normal inclination of the incisors.

Although these thresholds provide useful biomechanical guidelines for clinical practice, they should be interpreted as theoretical references rather than direct clinical recommendations, given the model assumptions and biological variability. Importantly, the present FE results are consistent with clinical outcomes reported in the literature, such as Lin et al. [15], who demonstrated that mini-screw anchorage can effectively reinforce molar anchorage and improve torque control and incisor intrusion during space closure with clear aligners.

Moreover, the material properties of aligners may substantially influence the magnitude of force required. Softer materials with greater stress relaxation may achieve the intended tooth movement with relatively smaller forces, whereas stiffer materials with less relaxation may require larger forces to produce the same movement. Thus, the optimal loading conditions suggested by this FE model should be interpreted in the context of material variability, and future clinical studies are warranted to verify these thresholds under different material conditions and patient-specific scenarios.

## Data Availability

No datasets were generated or analysed during the current study.

## Abbreviations

FE: Finite element
3D: Three-dimensional
